# Disk Harmonic Mapping of Cranial Surfaces for Fracture Visualization

**DOI:** 10.1109/OJEMB.2026.3681346

**Published:** 2026-04-06

**Authors:** Nicolas Hadjittoouli, Costas Pitris

**Affiliations:** KIOS Research and Innovation Center of ExcellenceUniversity of Cyprus 2109 Nicosia Cyprus

**Keywords:** Biomedical imaging, computed tomography, computer-aided diagnosis, skull, visualization

## Abstract

*Goal:* Skull fractures, especially those involving the cranial base and facial regions, present significant diagnostic challenges due to the skull's complex anatomy and subtle radiographic findings. Accurate detection requires repeated and meticulous examination of multiple CT slices, which is a significant cognitive burden, and requires considerable interpretation time. The primary objective of this study is to develop a visualization flattening technique that effectively transforms the curved skull surface into planar representations that enhance fracture features. *Methods:* A novel visualization process was developed that extracted the cranial surface and subsurface layers from head CT scans and used disk harmonic mapping to generate flattened representations of the lower, upper, occipital, and frontal hemispheres of the skull. The technique was applied to nine cases from the CQ500 dataset, with varying levels of inter-reader agreement, or lack thereof, among the original radiologists who interpreted the dataset. These cases encompass both straightforward and diagnostically challenging fractures that exemplify the advantages of the proposed methodology. *Results:* The flattened views unwrapped the fractures into continuous, high contrast features, with improved conspicuity compared to the fragmented appearance across multiplanar reconstruction slices. Comparison with existing skull visualization methods, the proposed technique demonstrated high contrast of fractures features, and delineation between emissary veins, with less distortion and high preservation of anatomical continuity. *Conclusions:* Disk harmonic flattening offers a new approach to skull fracture visualization, providing radiologists and emergency department staff with a valuable addition to the conventional radiological tools, particularly in diagnostically challenging cases.

## I. Introduction

Skull fractures represent a significant portion of adult trauma, accounting for 36 of all cases [1]. Skull fractures can pose substantial diagnostic challenges, particularly those of the frontal and basilar regions of the skull. Misdiagnosis of fractures in these anatomically complex areas can lead to severe complications and potentially life-threatening consequences for the patients. Among the critical sequelae associated with undetected skull fractures are vascular intracranial and orbital injuries, cerebrospinal fluid (CSF) leakage, and cranial nerve palsies [2], [3]. Furthermore, studies indicate that approximately 1030 of patients with skull base fractures subsequently develop CSF fistulae [3], [4], [5], [6], [7], [8], [9].

Fractures affecting the cranial base and facial region are particularly challenging for both the diagnosis and management of head trauma [2], [7], [10]. Hence, radiologists conventionally employ a methodical approach to evaluate head computed tomography (CT) scans, systematically navigating through axial, sagittal, and coronal planes to mentally reconstruct the complex three-dimensional architecture of the skull. Currently, multi-planar reformat assessment combined with windowlevel settings and maximum intensity projection (MIP) are the essential components of the radiologist's toolbox for the comprehensive evaluation and detection of the fractures. While these visualization techniques enable examination of osseous structures from multiple perspectives, the process becomes particularly labor-intensive when analyzing the more anatomically complex regions of the cranium.

The identification of subtle pathological findings frequently depends on the radiologist's clinical experience, introducing potential variability in diagnostic outcomes. More so, the effective diagnosis of skull fractures is challenging across the entire spectrum of radiological expertise, impairing the diagnoses of both residents in training and experienced practitioners, particularly in busy emergency care settings. Multiple studies have documented significant rates of misinterpretation in the evaluation of head CT scans for skull fractures [9], [11], [12], [13], [14].

A key contributor to this diagnostic difficulty is distinguishing skull fractures from other normal features. Cranial sutures, accessory sutural variants, emissary veins, and vascular channels can have similar radiographical appearance to fracture lines. Conversely, genuine fractures may be overlooked as benign sutural or vascular anatomy. This overlap increases the risk of both false-positive and false-negative interpretations, potentially leading to missed diagnoses. Fracture detection within the facial and basilar regions of the skull is also a significant challenge for Computer-Aided Diagnosis (CAD) systems [10], [13], [15]. The complex architecture and superposition of osseous structures in these anatomical areas frequently obscure fracture lines, hindering reliable detection [10]. Contemporary automated classification algorithms have not demonstrated adequate performance in addressing these specific anatomical regions, with the majority of research focusing on calvarial fractures instead [15], [16], [17], [18]. These diagnostic challenges underscore the need for improved methodologies to enhance detection.

Flattening of imaging volumes has gained significant attention in medical imaging applications since it can facilitate easier and more precise diagnosis [19], [20], [21], [22], [23], [24]. Several techniques can be utilized for this purpose, with the choice of approach depending mainly on the application and the anatomy under investigation. Each approach provides advantages and disadvantages with regards to feature preservation and computational complexity [21]. Flattening techniques have the potential to address two important challenges in the radiological evaluation of imaging volumes, i.e., the speed and accuracy of the radiological evaluation process.

In skull fracture diagnosis, the implementation and impact of curved-MIP for flattening and visualization have been demonstrated by Ringl et al. [19]. The resulting flattened views enabled inexperienced radiologists to achieve performance levels comparable to experts, while experienced practitioners were able to achieve substantially higher fracture detection rates [19]. However, that study employed a proprietary, undisclosed, algorithm. Visually similar results can be obtained using azimuthal equidistant mapping (AEM) projection [24]. Another approach is to employ computational volumetric deformation to flatten the cranial structures to anatomy driven reformations (ADR) [25]. ADR allowed radiologists to examine different flattened anatomies in multiplanar reformats. All the above methods rely on deriving relevant attributes (i.e., segmentation, surfaces, boundary) from the image data. The integration of differential geometry principles for planar visualization also facilitate enhanced diagnostic accuracy and efficiency [17], [18], [26], [27].

In this study, a novel approach was developed for the flattening of the skull architecture. Harmonic mapping was employed to transform the cranial CT scans into a flattened topological unit disk. In addition, surface parametrization of the skull was used to derive the attributes necessary, rather than using the anatomical assumptions utilized by other techniques. A unique and critically important characteristic of the proposed approach is that using disk harmonic mapping from surface mesh representation preserved critical skull anatomical features and the skull's intrinsic geometric properties during the deformation. In addition, this technique ensures that the constructed flat surface maintains the continuous smoothness of osseous structures, without edge discontinuities, thereby preventing singularity formation during the transformation process. These characteristics result in a marked improvement over the proprietary images in the literature [19] as well as AEM [24] techniques, which can introduce substantial distortions to global geometric structures. The proposed approach results in superior and significantly more interpretable flattened visualizations compared to other approaches.

The publicly available CQ500 dataset was used in this study. In this dataset, each case was evaluated by three independent radiologists with different experience levels [16]. In this dataset, 84 cases were reported as fractures by at least one radiologist, while only 14 received unanimous agreement across all three readers, a sixfold discrepancy that highlights the subjective nature of skull fracture interpretation on conventional CT and motivates the need for enhanced visualization techniques (Supplementary Material, Table I). Statistical analysis of the radiological diagnosis, for the cases of skull fractures, revealed poor agreement in the intraclass correlation coefficient [24] (Supplementary Materials, Materials and Methods, A. Dataset).

## II. Materials and Methods

**Fig. 1.**
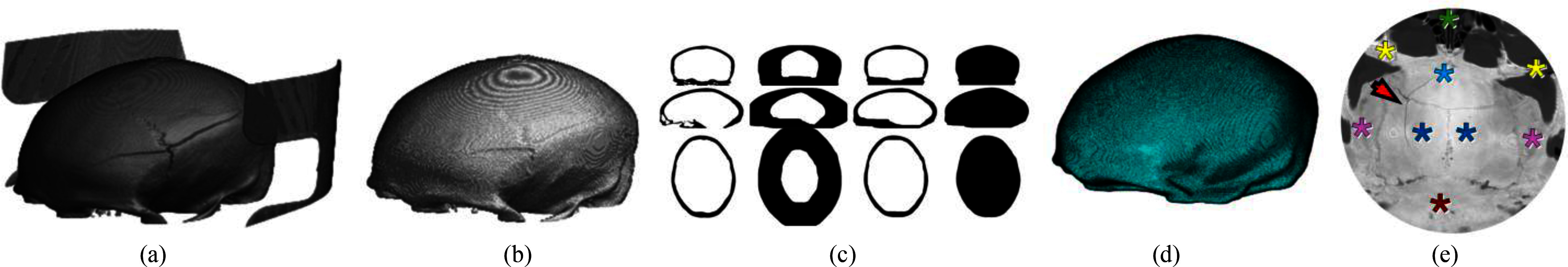
Pipeline for flattening of a scaphocephaly skull of Patient 13 from CT scan (a) to the planar view (e). Pre-processing stages (a-c) extract the point cloud: (a) Gaussian blurring and thresholding, (b) largest connected component, (c) coronal (top row), sagittal (middle row), axial (bottom row) views, and each column shows dilation, erosion and filling operations performed along the axial axis to trace the outer boundary of the skull. (d) Alpha-shape algorithm generates the skull mesh using the minimum alpha value ( critical) that encloses all points of the skull. (e) Disk harmonic mapping flattens the mesh to a unit disk. Red arrow indicates the fracture. Regional color-coded landmarks () denote cranial bones: ethmoid (green), zygomatic (yellow), frontal (cyan), parietal (blue), temporal (pink), and occipital (brown) bones. In this case all 3 radiologists have correctly identified the fracture.

The objective of this study was to provide interpretable flattened visualizations of the skull, including the diagnostically challenging base and face regions. In addition, going well beyond the current state of the art, the proposed technique also allows visualization of the internal bone structure, at various depth levels, for unprecedented subsurface evaluation. In summary, the skull bone, segmented from the CT scans, was transformed into a simply connected surface with an open boundary to define the watertight geometry of the skull, which is a mathematical prerequisite of the disk harmonic mapping (Fig. 1). In addition, isosurfaces at various depths within the bone were obtained. Using disk harmonic mapping, each isosurface was mapped and flattened to a unit disk allowing separate visualization of varying depths (Supplementary Materials, Materials and Methods, C. Isosurface Construction). The code in this study is publicly available at https://doi.org/10.5281/zenodo.19336059.

To project to the unit disk, initially, the Euclidean distance (1) of the boundary vertices was calculated to perform arc-length parametrization (2) of each boundary vertex. Subsequently, each boundary vertex $v$ was mapped to the unit disk in the complex plane, as described by (3), the Euler's formula (Supplementary materials, Fig. 1).

\begin{align*}
&{{s}_v} = |\left| {\mathop {{{v}_j}}\limits^{\rightarrow} - \mathop {{{v}_{j - 1}}}\limits^{\rightarrow} |} \right| \tag{1}\\
&{{\theta }_v} = 2\pi \frac{{{{s}_v} + {{s}_{v - 1}}}}{{\mathop \sum \nolimits_{k = 1}^n {{s}_k}}} \tag{2}\\
&{{z}_v} = {{e}^{i{{\theta }_v}}} \tag{3}
\end{align*}

**Fig. 2.**
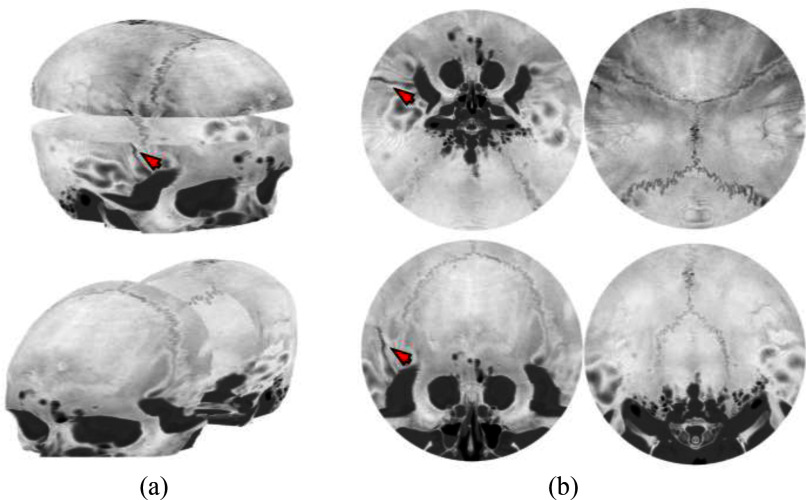
Skull mesh decomposition and flattening of trigonocephaly skull of Patient 134. (a) 3D skull mesh partitioned into LUFO regions (Lower, Upper, Front, Occipital) creating four simply connected open surfaces, each color-mapped with CT intensity values (HU). (b) Corresponding flattened representations obtained via disk harmonic mapping, preserving the geometric relationships of each surface in 2D planar space. Arrows indicate a fracture at the temporal bone. In this case only 1 out of 3 radiologists has correctly identified the fracture.

Using the Laplace cotangent matrix ($L$), formulated in (4), the linear system, expressed in (5), was solved to obtain the disk harmonic mapped coordinates ($z^{\prime}$). The constrain vector ($z$) is a null vector of the vertices, except for the boundary vertices, that correspond to the mapped boundary complex coordinates calculated in (5), known as the Dirichlet constraints (Supplementary Materials, Materials and Methods, Fig. 2 and D. Disk Harmonic Map).

\begin{align*}
L &= \left\{ \begin{array}{ll} {{w}_{ij}} = \frac{1}{2}\ \left( {cot\ {{\alpha }_{ij}} + cot\ {{\beta }_{ij}}} \right) & {\rm if}\ i\ne j\\
 {{w}_{ij}} = - \sum \nolimits_{j\in N\left( i \right)} {{w}_{ij}} & {\rm if}\ i = j\\
 {{w}_{ij}} = 0 & {\rm otherwise} \end{array} \right. \tag{4}\\
z^{\prime} &= {{L}^{ - 1}}z \tag{5}
\end{align*}

To create the inner isosurfaces, first, the unit normal vector of each vertex was computed. Surfaces were created by combining the points perpendicularly below the outer surface at each given distance. The number of isosurfaces created depended on the thickness of the skull, and the spacing between the isosurfaces, was set as the minimum dimension of the voxel size (Supplementary Material, Materials and Methods, C. Isosurface Construction).

Mapping from three-dimensions to two-dimensions unavoidably introduces distortion [30]. To evaluate level of the distortion and the geometric reliability of disk harmonic maps, angle and area distortion metrics were computed. Two vectors from each facet $( f )$ were selected to apply cosine similarity (6) and cross product (7). The angular absolute difference (8) and the area logarithmic ratio (9) were employed to evaluate the mapping's geometric distortion, respectively.

\begin{align*}
&{{\theta }_f} = {{\cos }^{ - 1}}\left( {\frac{{{{u}_f}\ {{v}_f}}}{{\|{{u}_f}\|\ \|{{v}_f}\|}}} \right) \tag{6}\\
&{{A}_f} = \frac{{{{u}_f} \times {{v}_f}}}{2} = \frac{1}{2}\left| {\begin{array}{ccc} \bm{i}&{\bm{j}}&{\bm{k}}\\ {\begin{array}{c} {{{u}_1}}\\ {{{v}_1}} \end{array}}&{\begin{array}{c} {{{u}_2}}\\ {{{v}_2}} \end{array}}&{\begin{array}{c} {{{u}_3}}\\ {{{v}_3}} \end{array}} \end{array}} \right| \tag{7}\\
&{{E}_\theta } = \left| {\theta {{^{\prime}}_f} - {{\theta }_f}} \right| \tag{8}\\
&{{E}_A} = \log \left( {\frac{{{{{A^{\prime}}}_f}}}{{{{A}_f}}}} \right) \tag{9}
\end{align*}

## III. Results

**Fig. 3.**
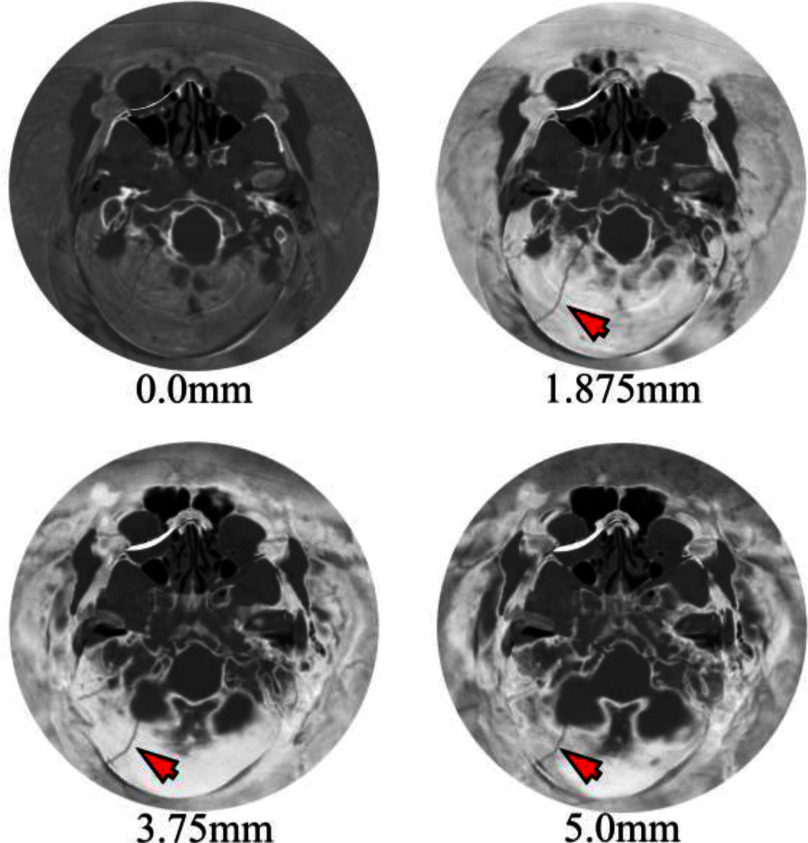
Isosurface-by-isosurface examination of Patient 383 showing an occipital fracture. Projection of eight sequential depth levels, progressing from the outer skull surface (0mm) through the bone thickness until 5mm of depth, revealing the fracture extent across multiple layers. Arrows indicate fracture locations. In this case only 1 out of 3 radiologists has correctly identified the fracture.

**Fig. 4.**
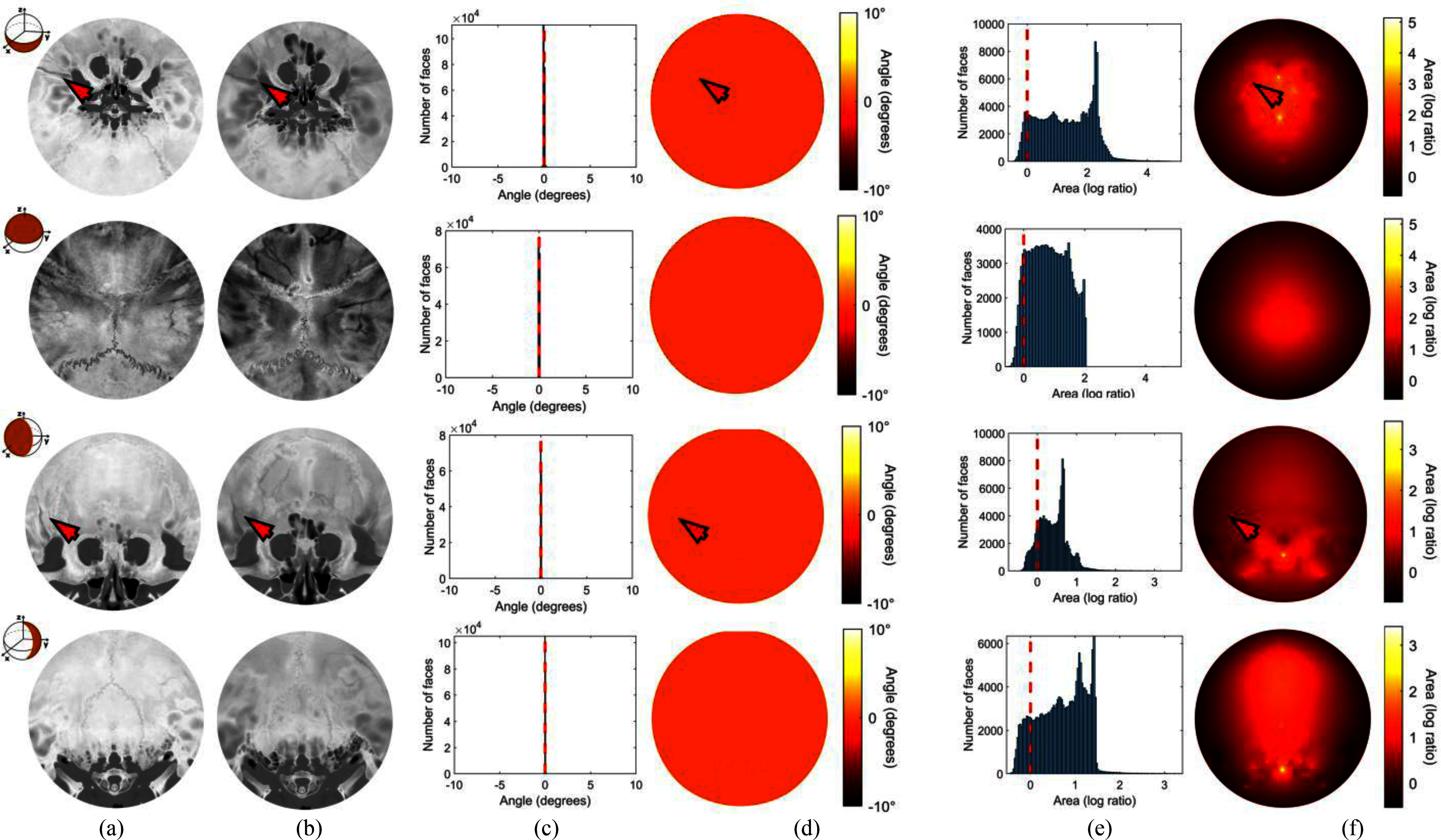
Evaluation of the disk harmonic map for Patient 134 as (a) maximum intensity projection, and (b) average intensity projection. Angular distortion is illustrated in (c) the histogram of the mean of angle deviations, and (d) the equivalent quasi-conformal visualization of the distortion, computed using the cosine similarity for each facet. Similarly, the area distortion is depicted in (e) the histogram distribution and (f) the equivalent area distortion visualization, computed using cross product for each facet. Arrows indicate fracture locations. Horizontal dotted lines represent the undistorted value. Note that, although the fracture is reaching the boarder in the Lower hemisphere, it appears as continuous in the Frontal projection.

The constructed simply connected open surface served as the input to the disk harmonic mapping that projected the three-dimensional structure to a two-dimensional plane. The surface of the skull was divided into LUFO regions to produce visualizations from four different perspectives, and the intensity Hounsfield Units (HU) values were interpolated to the vertex colors of each surface. Sample results are demonstrated in Fig. 2 for patient 134. An overlap of 10 is applied between adjacent LUFO regions sharing a boundary, ensuring that fractures near boundary zones are visible in both neighboring views. The unit normal vectors of the vertices and facets were used to generate the additional subsurface isosurfaces. Fig. 3 illustrates four consecutive isosurfaces for patient 383. The patient suffered an occipital fracture whose extend along the depth of the skull bone can be evaluated in these subsurface views. The patient in Fig. 4 (patient 134) is used to demonstrate the performance of the proposed method in terms of projection quality and geometric distortion characteristics. Visualizations of the average and maximum intensity projections (AIP and MIP) along with their corresponding distortion maps and distortion distributions are depicted. Angle distortion is less than 2 degrees and area distortion is below 2 orders of magnitude, indicating the mapping is quasi-conformal with highly preserved local geometric relationships.

The computational complexity of solving the harmonic map is O(n^3^) for dense systems. However, by exploiting the sparsity of the cotangent Laplacian matrix reduces the complexity to O(n^2^). The pipeline ran for 16.97 seconds on an Intel i7-10700 CPU with 8 cores of 2.90GHz and 16GB of RAM.

## IV. Discussion

The proposed flattened visualization enhances the visibility and increases the contrast of complex fractures in CT images by constructing a tangent perspective of the skull, where fractures are perpendicular to the bone's surface. Most importantly, it improves visibility of subtle fractures in the frontal and basilar regions, where there is significant diagnostic discrepancy, aiding in bridging the gap between experienced and inexperienced radiologists especially under the pressure of emergency department evaluations [19]. Another key advantage of this new approach is the ability to examine the skull in depth, isosurface-by-isosurface, allowing for an effective analysis of each subsurface layer to reveal subtle diagnostic features at different depths (Fig. 3). In addition, the LUFO projection scheme has been introduced (Fig. 2). This representation enhances the clinical utility of disk harmonic maps by exploiting the perpendicular axes of symmetry of the skull and offers clinicians intuitive and anatomically relevant views of fracture-prone zones. Moreover, it avoids fracture discontinuity. Since the LowerUpper and FrontalOccipital pairs are oriented orthogonally, a fracture that lays on the boundary in one pair of views will remain fully continuous in the complementary pair, and vice versa. This ensures that no fracture is simultaneously disrupted across all four representations (Supplementary Materials, Fig. 4). It also maintains anatomic consistency with minimal area distortion, without any obscuration by other bone structures, such as the zygomatic and ethmoid bone in the frontal views (Fig. 4).

**Fig. 5.**
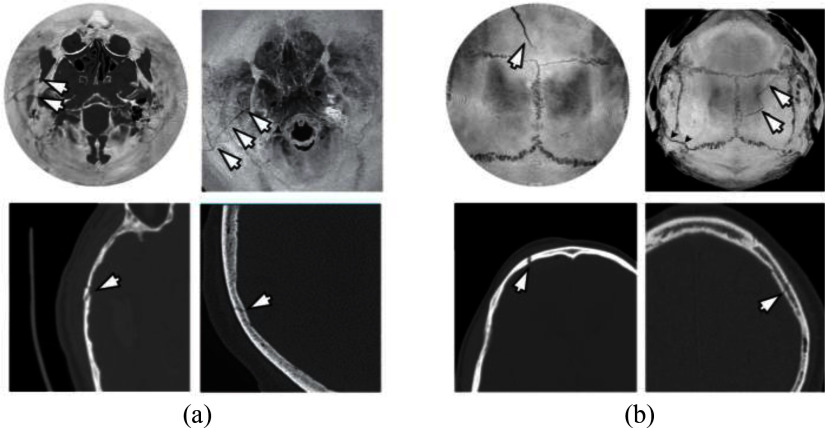
Comparison of disk harmonic mapping (left) versus skull unfold [19] (right) for visualizing (a) temporal (Patient 449) and (b) calvarial (Patient 137) fractures. Top: flattened representations. Bottom: corresponding axial CT slices magnified views of the fractured regions. Arrows indicate fracture locations. One radiologist missed the fracture in (a) whereas all 3 identified the one in (b).

**Fig. 6.**
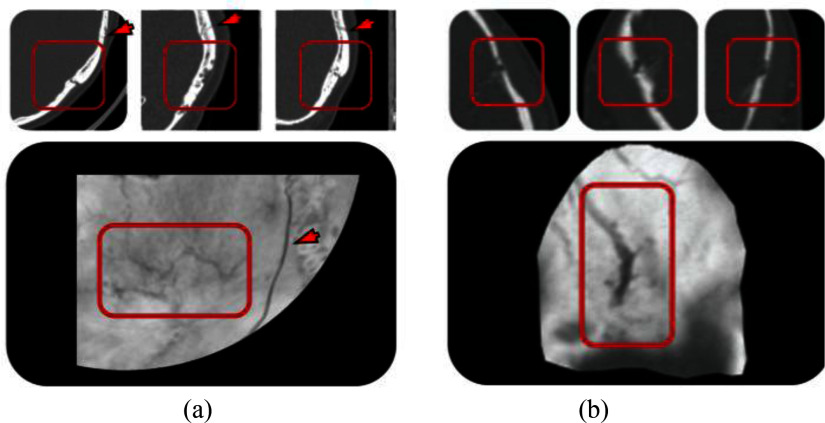
Comparison of emissary veins versus fracture in (a) disk harmonic mapping for Patient 248 and (b) ADR [25]. Top: Multi-planar reformats (axial, sagittal, coronal) CT slices magnified views of the abnormality regions. Bottom: corresponding flattened views. The rectangles indicate the emissary veins. Arrows indicates the fracture location. The fracture in (a) was missed by 2 out of 3 radiologists.

**Fig. 7.**
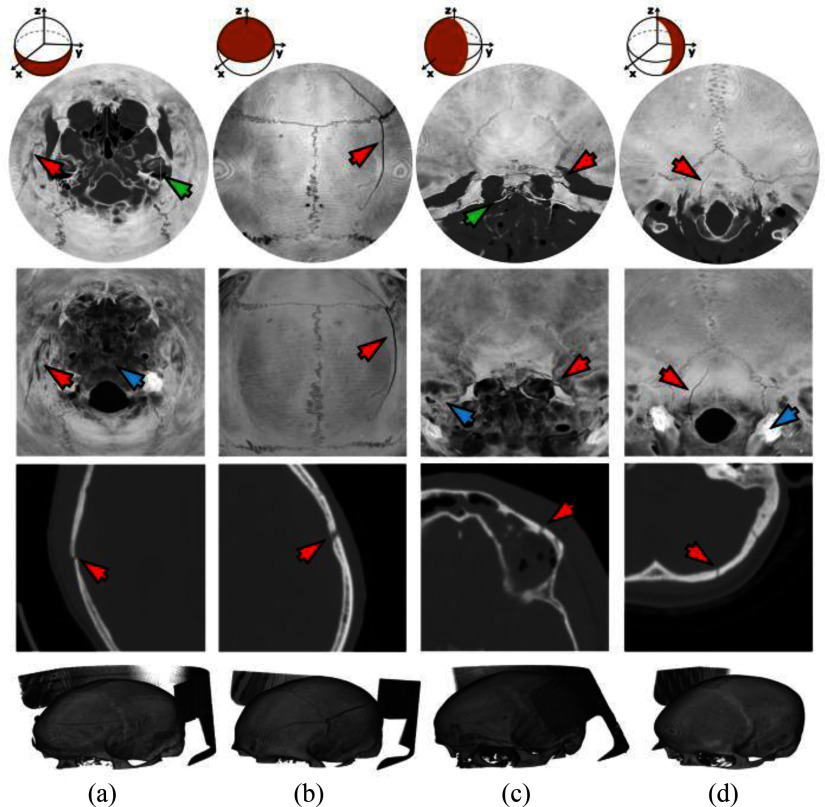
Comparative visualization of skull fractures in four patients, displaying different fractures, at their corresponding skull quadrant region: (a) Patient 107-Lower, (b) Patient 13-Upper, (c) Patient 241-Frontal, and (d) Patient 205-Occipital hemisphere projection. First row: disk harmonic mapping MIP, second row: AEM MIP [24], third row: magnified regions of axial CT slices, and fourth row: DICOM 3D reconstructions. The arrows indicate fractures (red), imaging artifacts (green), and obscured regions (blue). The fracture of Patient 107 was missed by one radiologists. Patients 13 and 241 were correctly diagnosed by all three whereas the fracture of Patient 205 was missed by 2 out of 3 radiologists.

Using the proposed technique, landmarks are easier to identify compared to other approaches proposed in the literature [19], [24]. These advantages are evident in comparison with H. Ringl et. al. [19] for similar fractures and flattening regions in the basilar region of the skull (Fig. 5). Furthermore, the anatomical features of the intricate structures, such as the osseous fragility, of the base of the skull are significantly clearer and distinct using the proposed visualization (Fig. 6). The comparison between disk harmonic mapping and the ADR method also reveals notable differences. Patient 248 demonstrates the difference between a skull fracture and emissary veins features and osseous fragility leading to false positive cases. Using the ADR method (Fig. 6(b)) the presence of an emissary vein leads to inconclusive evaluation and further investigation is recommended by the ADR method's authors. Therefore, using the proposed approach the fracture characteristics become more clearly discernible, as confirmed by their corresponding axial CT slices. In comparison with AEM (Fig. 7) [24], the new approach introduces considerably less distortion since it avoids radial stretching. In addition, it displays overlapping anatomical structures much more clearly. The reduction in the distortion is particularly important in peripheral regions of the skull, where fractures are often difficult to visualize due to the complex curvature. The disk harmonic method benefits are particularly evident in the frontal, lower, occipital regions of Fig. 7, where no obscuration is present (blue arrows). Another advantage, both in terms of computational burden and reduction in distortion, is that the proposed approach does not require precise orientation of the skull, using image registration, which can introduce further distortion due to misalignment. Minor artifacts are apparent in acute angles (green arrows, Fig. 7) as a result of the parametrization process. A more direct and thorough quantitative comparison with existing skull flattening methods was not feasible, as none of the three skull flattening techniques report distortion metrics. To the best of our knowledge, the per-patient angle and area distortion analysis presented in this work is the first to be reported for skull flattening.

Traditional intensity projections across multiplanar reformats along with AEM [24] present skull structures as overlapping layers in two-dimensional views, causing major obscurations from layer overlap that complicate fracture detection. Isosurface visualization at different depths eliminates the obscuration and ensures that the intricate geometry and the internal structure of the bone of the skull can be examined with improved visualization of the fractures and other structural details (Fig. 3). This stratification not only resolves the problem of overlapping structures but has the potential to enhance diagnostic precision, specifically in applications of automated skull fracture detection in CAD systems [19].

High resolution surface is crucial for an effective planar mapping, and errors such as holes or disconnected components lead to flawed maps. The proposed segmentation approach eliminates noise (e.g., bed, head holder) and artifacts to ensure a watertight outer surface of the skull (Supplementary Materials, Materials and Methods, B. Skull Boundary and Discussion). In the future, incorporating deep learning segmentation models can further improve the process [31]. A multi-class semantic segmentation of the skull bone can enhance the proposed approach by leveraging morphological insights to scale the osseus thickness for each region of the cranium. Such models could also address complex anatomies, including the foramina and the mastoid in the skull base region, which remain a visualization challenge shared with conventional CT. The alpha shape is sensitive to data quality and can produce non-manifold edges, thus, the preceding segmentation and morphological operations mitigate this by ensuring a connected point set, and loop subdivision [31] further ensures curvature continuity. Similarly, the accuracy of the subsurface layer generation is dependent on the constructed outer surface, and in regions with variable thickness, such as the mastoid, the normal-vector projection may not capture the internal bone trajectory adequately.

The benefits of flattened visualization can be very important in clinical settings and emergency departments, where visualization impacts the time and precision of diagnosis and subsequent treatment planning [19], [24], [25]. The main advantage of flattening techniques is that radiologists should be able to deliver a diagnosis just by looking at the maximum andor average intensity projection. Isosurface-by-isosurface examination (Supplementary Materials, Fig. 3) is only necessary if there is ambiguity or the fracture is challenging to discern. However, the diagnostic advantages of the proposed approach must be proven under rigorous clinical validation. Future studies will assess accuracy and inter-observer variability, prior to widespread adoption in clinical settings. Ideally, evaluators with varying levels of expertise would be randomly assigned to two groups to assess skull fractures using the CQ500 dataset. The first group would review half of the dataset using disk harmonic mapping and the remaining half using multiplanar reconstructions. The second group would evaluate the same cases but with the imaging methods reversed.

## V. Conclusion

In this study a new approach for the visualization of skull fractures is proposed. It addresses some of the limitations of the current visual-based radiological diagnostic approach applied to the skull, such as obstruction, distortion, inefficient projection of fine structures, and the inability to perform meaningful slice-by-slice examination in flattened visualizations. The effectiveness of the approach was demonstrated by applying the disk harmonic mapping technique to head CT scans from the CQ500 dataset for qualitative comparisons with other methods. The proposed system conformally flattens the skull surface, supports isosuface-by-isosurface examination, enhances projection of fine structures, and improves skull fracture features. Disk harmonic mapping is a promising technique for visualizing skull geometries, and it has the potential to become a valuable attribute in skull fracture detection by radiologists or CAD systems based on the experience of the Skull unfold study [19], [24], [25]. In radiologic interpretation, enhanced visualization is notably important, as radiologists predominantly rely on visual comparison for their diagnostic assessments [10], [11], [12], [13], [14].

## Supplementary Materials

The Supplementary Materials contain additional details of the dataset selection, the proposed pipeline pre-processing steps and distortion metrics, a complete set of results and further discussion.

Supplementary Materials
