# Supplementary material for: Disk Harmonic Mapping of Cranial Surfaces for Fracture Visualization
Source: IEEE Open J Eng Med Biol. 2026 Apr 6;7:139–45. doi: 10.1109/OJEMB.2026.3681346 (PMC13175593; doi:10.1109/OJEMB.2026.3681346)
Supplement: Supplementary Materials [file supp1-3681346.pdf]

Supplementary Materials

Disk Harmonic Mapping of Cranial Surfaces for Fracture Visualization

Nicolas Hadjittoouli, *Student member, IEEE* and Costas Pitris, *Member, IEEE*

I. Introduction

Fractures affecting the cranial base and facial region are particularly challenging for both the diagnosis and management of head trauma [1]. Hence, radiologists conventionally employ a methodical approach to evaluate head computed tomography (CT) scans, systematically navigating through axial, sagittal, and coronal planes to mentally reconstruct the complex three-dimensional architecture of the skull. Currently, multi-planar reformat assessment combined with window/level settings and maximum intensity projection (MIP) are the essential components of the radiologist's toolbox for the comprehensive evaluation and detection of the fractures. While these visualization techniques enable examination of osseous structures from multiple perspectives, the process becomes particularly labor-intensive when analyzing the more anatomically complex regions of the cranium.

Intensity projections, such as MIP, have emerged as a useful tool in medical imaging analysis for the assessment of images generated by various modalities, including magnetic resonance imaging (MRI), CT, positron emission tomography (PET), and ultrasound. MIP produces contrast-enhanced visualizations that significantly improve feature visibility, boosting diagnostic confidence, while enabling time-efficient assessments [2].

In this study, a novel approach was developed for the flattening of the skull architecture. Harmonic mapping was employed to transform the cranial CT scans into a flattened topological unit disk. In addition, surface parametrization of the skull was used to derive the attributes necessary, rather than using the anatomical assumptions utilized by other techniques. A unique and critically important characteristic of the proposed approach is that using disk harmonic mapping from surface mesh representation preserved critical skull anatomical features and the skull's intrinsic geometric properties during the deformation. In addition, this technique ensures that the constructed flat surface maintains the continuous smoothness of osseous structures, without edge discontinuities, thereby preventing singularity formation during the transformation process. These characteristics result in a marked improvement over the proprietary images in the literature [2] as well as azimuthal equidistant mapping [3] techniques, which can introduce substantial distortions to global geometric structures. The proposed approach results in superior and significantly more interpretable flattened visualizations compared to other approaches.

TABLE I
Diagnostic Discrepancies of Skull Fractures in the CQ500 Dataset

Level of agreement	✓	✓✓	✓✓✓	***Total***
Diagnosed patients	45	25	14	84

Distribution of diagnostic discrepancies among the radiologists, for the 84 patients with skull fracture that was confirmed by at least one radiologist in the dataset. ✓represents the number of radiologists that independently diagnosed the patient. [3]

TABLE II
Intraclass Correlation Coefficient of CQ500 Dataset

Samples	*ALL PATIENTS*		*SKULL FRACTURES*	
F-statistic	3.549		1.548	
p-value	<0.001		0.025	
df1	490		57	
df2	980		114	
Raters	*Absolute agreement*	*Conci-stency*	*Absolute agreement*	*Conci-stency*
ICC (95% CI)	0.444 (0.38-0.5)	0.705 (0.65-0.75)	0.082 (0-0.22)	0.0211 (0-0.45)

Absolute agreement and consistency using the two-way random effects method intraclass correlation between the radiologists in all (491 cases) and skull fracture patients (84 cases) of the CQ500 dataset [3].

II. Materials and Methods

A. Dataset

The publicly available CQ500 dataset was used in this study. It consists of 491 computed tomography (CT) scans and includes multiple interpretative assessments, with ethics committee approvals waiving informed consent. Each examination contains anonymized DICOM files accompanied by three independent radiological evaluations, performed by experienced practitioners with 8, 12, and 20 years of clinical expertise in cranial CT interpretation [4]. These radiological assessments were conducted according to standardized evaluation protocols. Within the CQ500 dataset, 84 patients were identified as having skull fractures by at least one radiologist. Of the 84 patients, only 14 (16.7%) were unanimously identified as having skull fractures by all three radiologists (Table I). Statistical analysis of those cases revealed poor agreement in the intraclass correlation coefficient (Table II) [3]. These findings quantitatively illustrate the inherent diagnostic challenges associated with skull fracture identification and underscore the influence of the radiologist's experience on diagnostic accuracy. Three of the cases selected are confirmed fractures, and six cases were randomly selected from the diagnostic disagreement cases (Supplementary Material, Table III) to (i) enable direct comparison with

TABLE III
DISCREPANCY AGREEMENT FOR THE CASES IN THIS STUDY

Patient code	*Radiologist 1*	*Radiologist 2*	*Radiologist 3*
13	✓	✓	✓
107	✓	✓	–
134	✓	–	–
137	✓	✓	✓
205	✓	–	–
241	✓	✓	✓
248	✓	–	–
383	✓	–	–
449	✓	✓	–

Agreement in the radiologists' diagnosis of the selected nine cases from the CQ500 dataset, where ✓ and – indicate the positive and negative presence of fracture each case, respectively. [4]

previously reported skull flattening methods and (ii) demonstrate the proposed method's capacity to enhance fracture features in cases where conventional CT interpretation led to inter-observer diagnostic discrepancies.

B. *Skull Boundary*

The bone in the CT volume was segmented by applying Gaussian blurring and threshold with a filter size of 3, standard deviation of 1, and threshold set at +1100 Hounsfield Units (HU) with window width of 400 HU [5]. Although this process created a binarized volume of the bone, it did not eliminate all the noise. The connected components method [6] was then used to select the largest continuous volume. A pair of adjoined voxels of the same value were considered to belong to the same connected component if they shared a face. The largest connected object was assumed to be the bone of the skull. However, the boundary of the segmentation was non-continuous, which could lead to holes in the resulting surface. To create a suitable, simply connected surface, the interior of the skull was filled before extracting the outer boundary. Each axial slice was filled, with a pixel seed set at the center of the slice. This process could not be implemented at the base of the skull due to bone discontinuities. For those slices, dilation and erosion relaxations [7], with a filter size of 50, were applied to connect neighbor pixels (Fig. 3c-d). The resulting structure was a point cloud derived from the segmentation of the skull with the boundary vertices defined by the outer surface of the bone.

C. *Isosurface Construction*

The first step in the isosurface construction was to obtain the triangulation of the outer surface of the skull as a simply connected open surface. The alpha shape method was employed to establish the vertices' connectivity [8]. It provided a parameterized generalization of the convex hull for a discrete point cloud, enabling the reconstruction of non-convex shapes. Given a finite set of points, the Delaunay triangulation was computed, which partitioned the convex hull of the point cloud into simplices with the condition that no point lied inside the circumsphere of any simplex controlled by the α value. By adjusting the α value, the overall surfaces details could be adjusted from fine to coarse. The ***α-critical*** value was selected, which is defined as the smallest α value that encloses all the points [8].

Subsequently, using the set of points at the boundary, the alpha shape constructs the outer surface of the skull. The surface was further refined using loop subdivision [9]. The result was an ideally smooth surface, which is a critical requirement for disk harmonic mapping. Although projecting the entire skull to a unit disk is possible, the surface of the skull was divided into lower, upper, frontal, occipital (LUFO) regions to produce four simply connected open surfaces. The choice of four perspectives versus a single disk for the entire skull was to (i) allow direct comparison with previous works [3] and (ii) provide views that are easier for radiologists to visually interpret and to follow known radiological landmarks.

Using the set of the boundary's facets from the alpha shape, the outer surface of the skull was constructed. In addition, to create the inner isosurfaces, i.e. surfaces within the skull's bone, the unit normal vector ($\vec{n}$) of each vertex ($\vec{v}$) (1). The number of the produced isosurfaces (d) depended on the thickness (T) of the skull, and the spacing (m) between the iso-surfaces, obtained from the minimum dimension of the voxel size (2). Based on morphometric studies, the mean thickness of the frontal, parietal and temporal and occipital bone are 8.02±1.97mm, 7.04±1.43mm, 4.71±1.34mm and 7.98±2.47mm, respectively [10]. Therefore, the maximum thickness of T=10mm was used to produce isosurfaces that lie within the boundaries of the skull's bone.

$$\overrightarrow{v_{d+1}} = \overrightarrow{v_d} - m\,\vec{n} \tag{1}$$

$$d = \{u \in \mathbb{Z} \mid 0 \leq u \leq \tfrac{T}{m}\} \tag{2}$$

The surface was further refined using loop subdivision [9]. This approach offers two important advantages: (i) first derivative (C^1) and second derivative (C^2) smoothness are guaranteed and (ii) that is an approximating type of subdivision. Thus, loop subdivision preserved the topology of the shape, while simultaneously smoothing sharp edges into curves, thus enhancing the smoothness of the flattened view. The result was an ideally smooth surface, a critical requirement for disk harmonic mapping.

D. *Disk Harmonic Map*

Assuming Ω to be an arbitrary simply connected surface with boundary ∂Ω, a mapping $f\colon \Omega \rightarrow \mathrm{D}$ is obtained, where D is the unit disk, such that f is harmonic in the interior of Ω, while ∂Ω is mapped to ∂D by parameterizing the arc length of ∂Ω (Fig. 2). In the continuous case, the harmonic function of the scalar function (3) must satisfy the Laplace equation (4), which is equivalent to the discrete Laplace operator (5), calculated from the adjacent vertices $N(i)$ of each vertex.

$$f(x, y, z) = ax + by + cz \tag{3}$$

$$\nabla \cdot \nabla f = \Delta f = 0 \tag{4}$$

$$\Delta f(v_i) = \frac{1}{2A_i} \sum_{j \in N(i)} \left(\cot \alpha_{ij} + \cot \beta_{ij}\right)\left(v_i - v_j\right) \tag{5}$$

Initially, Euclidean distance (6) of the boundary vertices was calculated to perform arc-length parametrization (7) of each boundary vertex and map each boundary vertex v to the unit disk in the complex plane, described by the Euler's formula *(8)*.

$$s_v = || \vec{v_j} - \overrightarrow{v_{j-1}} || \quad (6)$$

$$\theta_v = 2\pi \frac{s_v + s_{v-1}}{\sum_{k=1}^{n} s_k} \quad (7)$$

$$z_v = e^{i\theta_v} \quad (8)$$

Where, z_v was the mapped complex coordinate, s_v was the length between each adjacent boundary vertex, and $\sum_{k=1}^{n} s_k$ was the total length of the boundary.

Using the Laplace cotangent matrix (L), which was constructed by (9) derived from (5), the linear system expressed in (10), was solved to obtain the disk harmonic mapped coordinates (X). To enforce the restraint of the boundary vertices, their diagonal element in L was set to one. Additionally, the constrain vector C is a null vector of the vertices, except for the boundary vertices that correspond to the mapped boundary complex coordinates calculated in (8).

$$L = \begin{cases} w_{ij} = \frac{1}{2}\left(cot\,\alpha_{ij} + cot\,\beta_{ij}\right) & if\ i \neq j \\ w_{ij} = -\sum_{j \in N(i)} w_{ij} & if\ i = j \\ w_{ij} = 0 & otherwise \end{cases} \quad (9)$$

$$z' = L^{-1} z \quad (10)$$

E. Distortion Metrics

Mapping from three-dimensions to two-dimensions unavoidably there is distortion. To evaluate the distortion and the reliability of disk harmonic maps, the angle and the area distortion metrics were computed. Two vectors from each facet (f) were selected to apply the cosine similarity and cross product values that provide angle and area distortion metrics, computed by (11) and (12), respectively.

$$\theta_f = cos^{-1}\left(\frac{u_f\, v_f}{\|u_f\|\,\|v_f\|}\right) \quad (11)$$

$$A_f = \frac{u_f \times v_f}{2} = \frac{1}{2}\begin{vmatrix} \boldsymbol{i} & \boldsymbol{j} & \boldsymbol{k} \\ u_1 & u_2 & u_3 \\ v_1 & v_2 & v_3 \end{vmatrix} \quad (12)$$

III. Results

In this section, the selected nine cases flattened representations are illustrated. The slices of the confirmed fracture regions are depicted in Fig. 3. Using the angle (11) and area (12) geometric distortion metrics, the deviation of each facet in the mapped and the original surface is computed, and to determine the comparison between the two surfaces, the absolute difference (13) and logarithmic ratio (14) were employed, respectively.

$$E_\theta = \left|\theta'_f - \theta_f\right| \quad (13)$$

$$E_A = \log\left(\frac{A'_f}{A_f}\right) \quad (14)$$

The maximum intensity projections (MIP) and average intensity projections (AIP) as well as their equivalent distortion maps and histograms of the lower, upper, frontal and occipital regions are depicted in Fig. 4.

IV. Discussion

The proposed flattened visualization enhances the visibility and increases the contrast of complex fractures in CT images by constructing a tangent perspective of the skull, where fractures are perpendicular to the bone's surface. Most importantly, it improves visibility of subtle fractures in the frontal and basilar regions, where there is significant diagnostic discrepancy, aiding in bridging the gap between experienced and inexperienced radiologists especially under the pressure of emergency department evaluations [2]. This representation enhances the clinical utility of disk harmonic maps by exploiting the perpendicular axes of symmetry of the skull and offers clinicians intuitive and anatomically relevant views.

A comprehensive evaluation of disk harmonic mapping across all cases confirms that the method is quasi-conformal, preserving local angles at the cost of area distortion (Fig. 4). The angular distortion histograms show that angle deviations remain tightly at the boundary, while the area maps reveal non-uniform scaling inherent to conformal parameterizations. Importantly, the distortion patterns are not influenced by the presence of fractures, indicating that the mapping treats fractured and intact regions with comparable geometric fidelity. This enables fracture analysis on the flattened domain without being confounded by mapping artifacts.

The alpha shape method is data sensitive, and it can result in non-manifold sharp edges, hence, the preprocessing morphological operations produces a connected pointset without noise. Another way of mitigating this effect involves employing the loop subdivision [9] for curvature continuity, i.e., smoothing, which also enhances the resolution in flattened views. While there were no significant issues with the CQ500 dataset, extreme segmentation errors may occasionally necessitate manual correction of the points of failure (e.g. bed, head holder, non-manifold edges), specifically adjusting the segmentation and the surface topology to achieve a simply connected open surface.

The benefits of flattened visualization can be very important in clinical settings and emergency departments, where visualization impacts the time and precision of diagnosis and subsequent treatment planning [2], [3], [11]. The main advantage of flattening techniques is that radiologists should be able to deliver a diagnosis just by looking at the maximum and/or average intensity projection. Isosurface-by-isosurface examination is only necessary if there is ambiguity or the fracture is challenging to discern. However, the diagnostic advantages of the proposed approach must be proven under rigorous clinical validation. Future studies will assess accuracy and inter-observer variability, prior to widespread adoption in clinical settings.

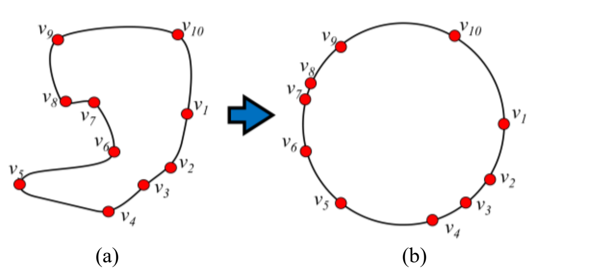

Fig. 1. Arc-length parameterization for boundary correspondence. (a) A discretized closed curve with vertices. (b) corresponding points mapped to the unit circle in the complex plane using Euler's formula (8) parameterized by cumulative arc-length (7), where normalized arc-length [0, 1] determines angular position [0, 2π].

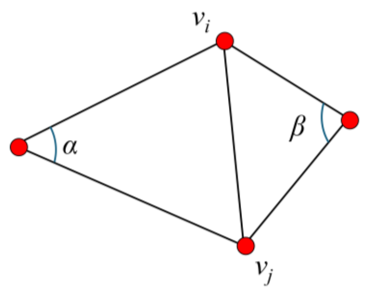

Fig. 2. Geometric construction of cotangent weights (w_{ij}) for discrete harmonic mapping. The angles α and β at the opposing vertices determine the edge weight between v_i and v_j via the cotangent formula (5) and (9).

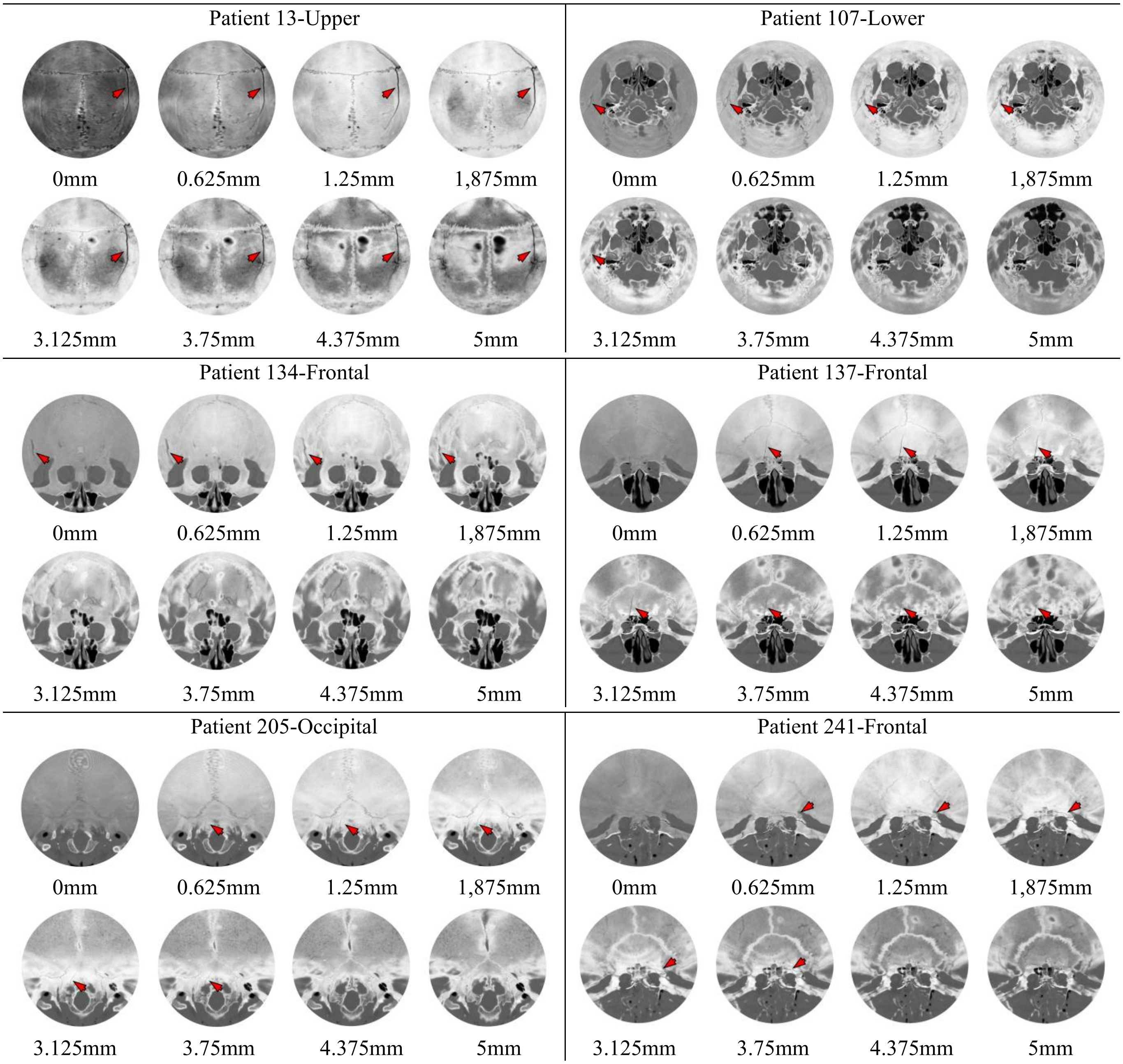

Fig. 3. (continues to the next page)

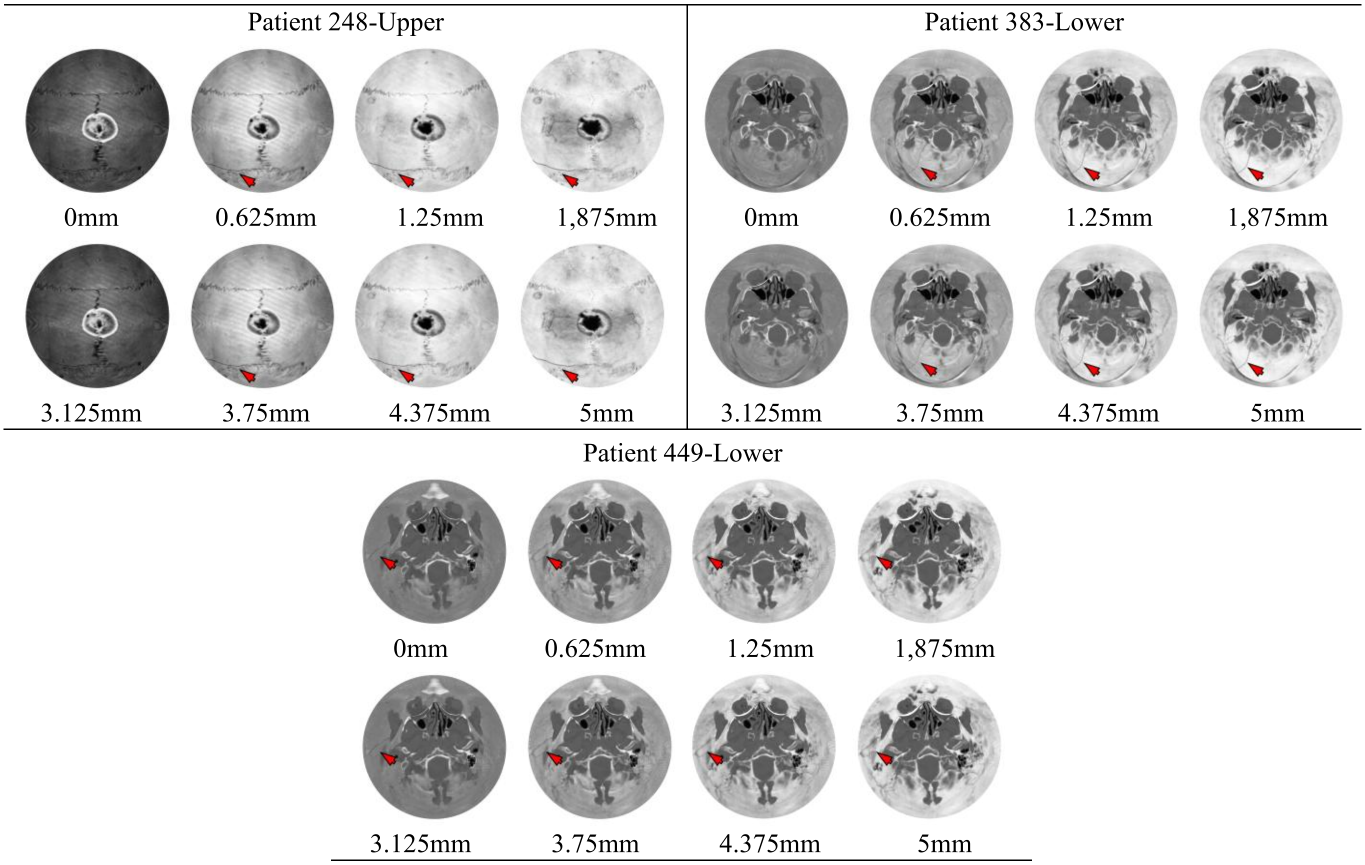

Fig. 3. Isosurface-by-isosurface slices of all nine cases showing the fractured region. Eight sequential depth levels progress from the outer skull surface (top-left) through 5mm of bone thickness (bottom-right), revealing fracture extent across multiple layers. Arrows indicate fracture locations.

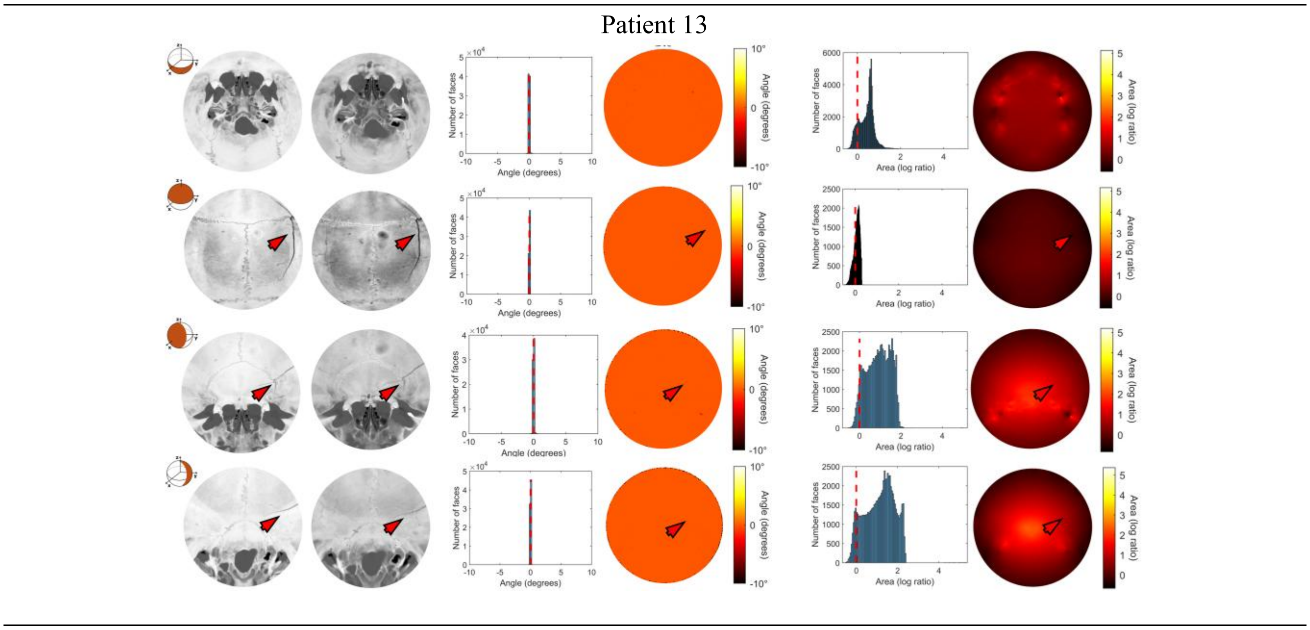

Fig. 4. (continues to the next page)

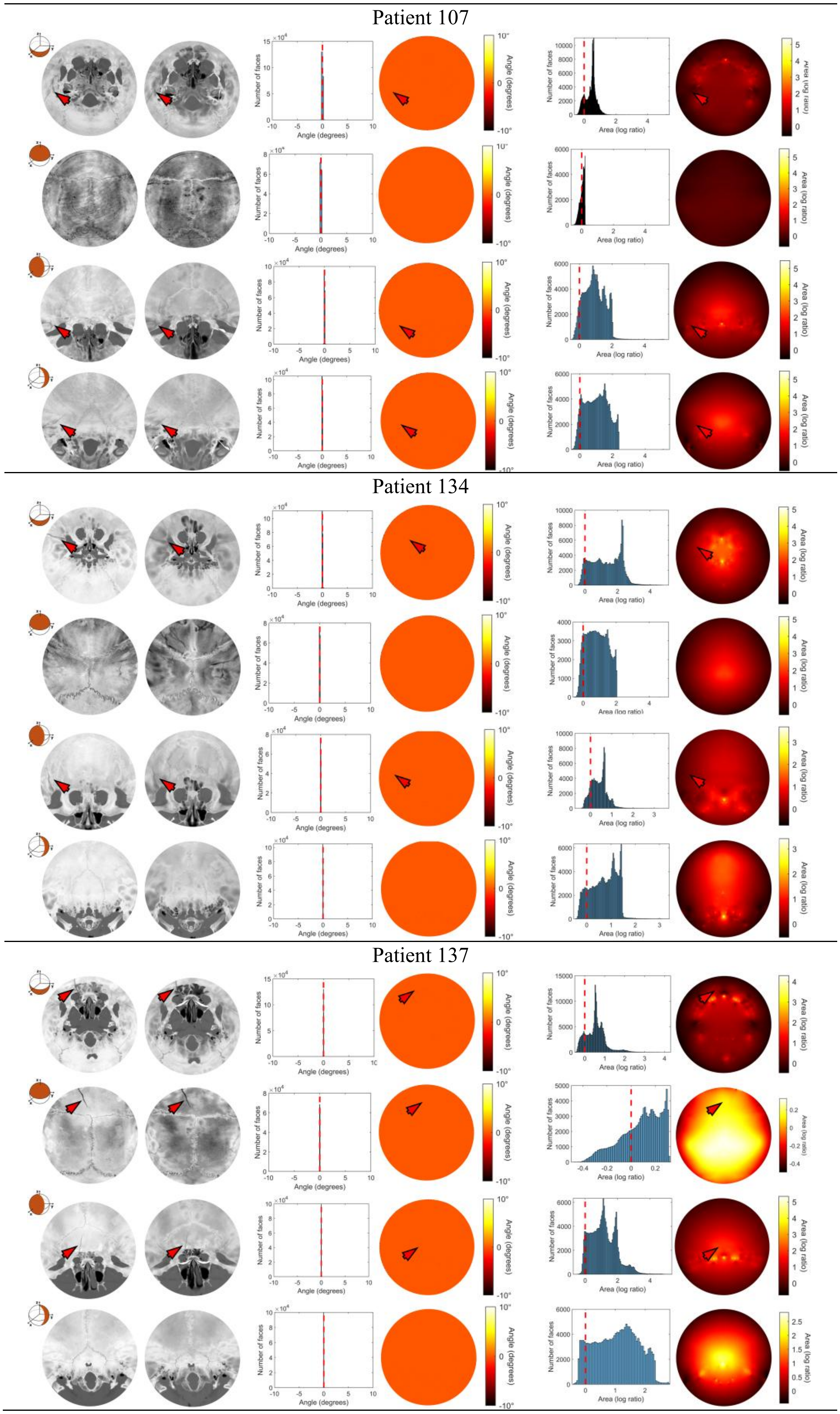

Fig. 4. (continues to the next page)

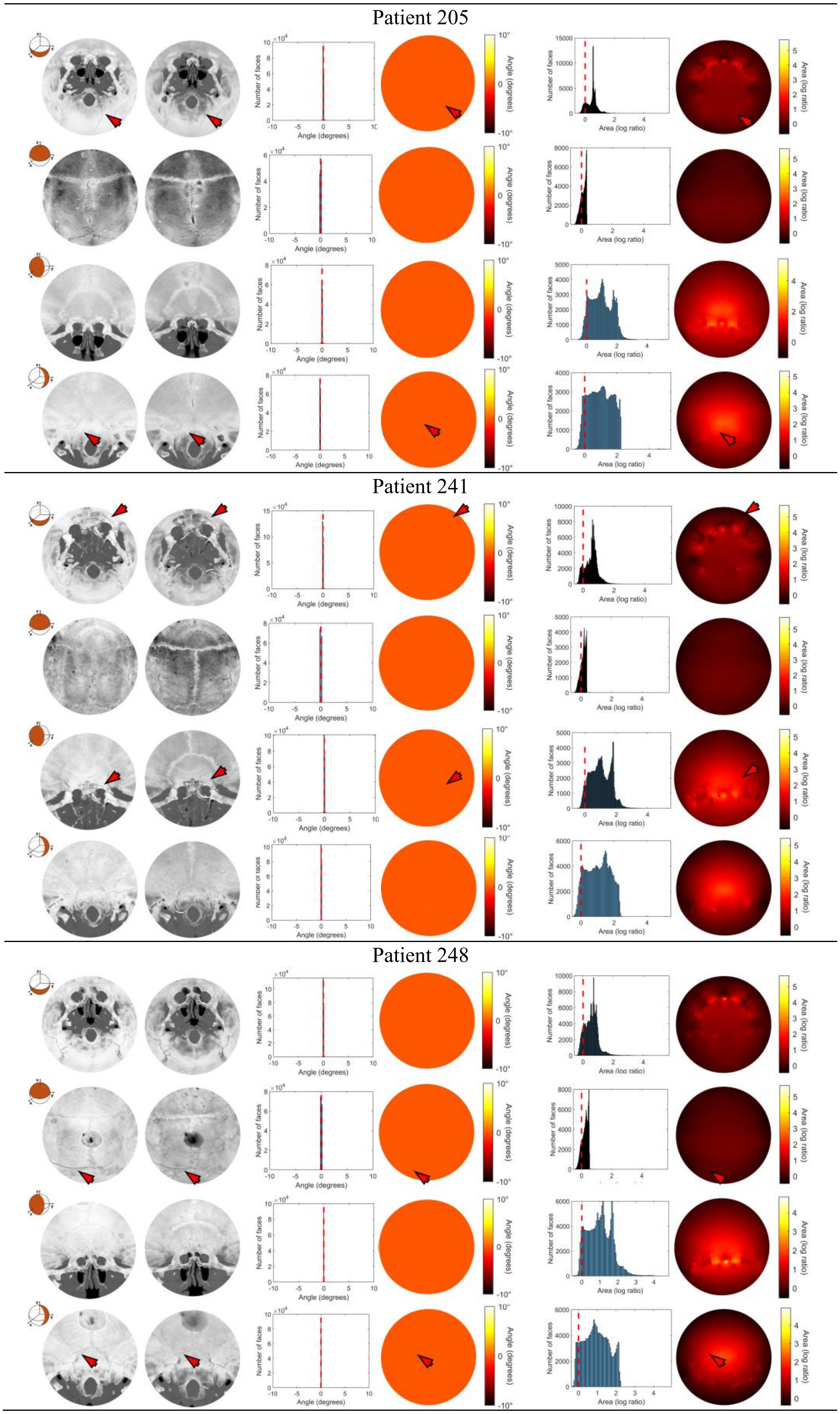

Fig. 4. (continues to the next page)

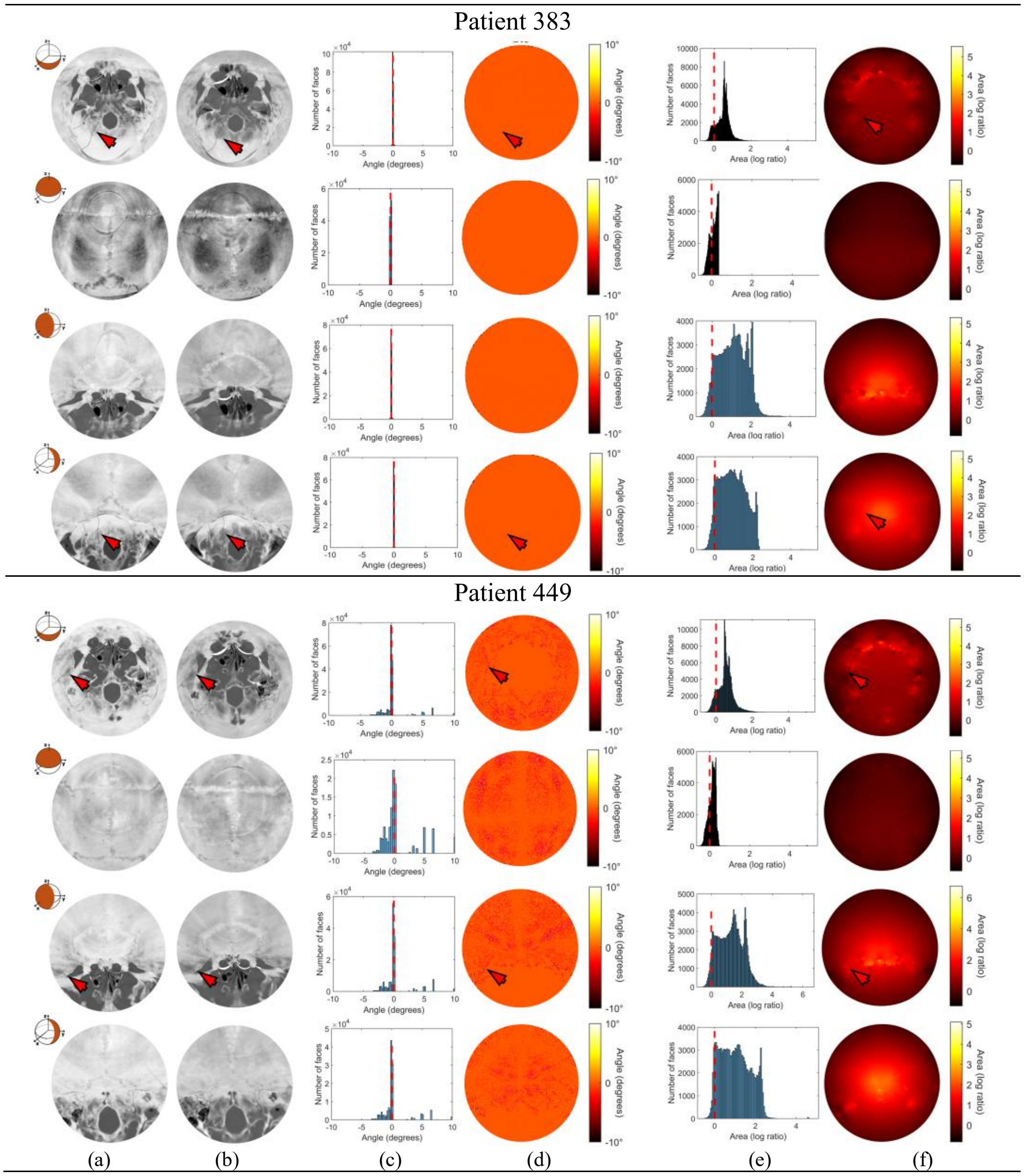

Fig. 4. Evaluation of the disk harmonic map of all nine cases in terms of (a) maximum intensity projection, (b) average intensity projection. Angular distortion validating that the method is conformal with (c) the histogram distribution of the mean of angle deviations, and (d) the equivalent quasi-conformal visualization computed using the cosine similarity for each face. Similarly, the area distortion is depicted in (e) the histogram distribution and (f) the equivalent area distortion visualization computed using cross product for each face. Arrows indicate fracture locations. Horizontal dotted lines represent the undistorted value.

V. References

[1] U. Perheentupa, A. A. Mäkitie, J. O. Karhu, P. Koivunen, R. B. Sequieros, and I. Kinnunen, "Frontobasilar fractures: Proposal for image reviewing algorithm," J. cranio-maxillo-facial Surg., vol. 42, no. 4, pp. 305–312, 2014, doi: 10.1016/j.jcms.2013.05.018.

[2] H. Ringl et al., "The skull unfolded: A cranial CT visualization algorithm for fast and easy detection of skull fractures," Radiology, vol. 255, no. 2, pp. 553–562, 2010, doi: 10.1148/radiol.10091096.

[3] N. Hadjittoouli, C. Nicolaou, and C. Pitris, "Azimuthal equidistant mapping and projection of head CT scans for fracture visualization," IEEE Access, vol. PP, p. 1, 2025, doi: 10.1109/ACCESS.2025.3565672.

[4] S. Chilamkurthy et al., "Deep learning algorithms for detection of critical findings in head CT scans: a retrospective study," Lancet, vol. 392, no. 10162, pp. 2388–2396, 2018, doi: 10.1016/S0140-6736(18)31645-3.

[5] M. G. F. Longo et al., "'All-in-one' window/level whole-body computed tomography scan – A faster way to evaluate trauma cases," Am. J. Emerg. Med., vol. 62, pp. 62–68, 2022, doi: https://doi.org/10.1016/j.ajem.2022.09.047.

[6] A. Rosenfeld and J. L. Pfaltz, "Sequential Operations in Digital Picture Processing," J. ACM, vol. 13, no. 4, pp. 471–494, Oct. 1966, doi: 10.1145/321356.321357.

[7] I. T. Young, "Image analysis and mathematical morphology, by J. Serra. Academic Press, London," Cytometry, vol. 4, no. 2, pp. 184–185, Sep. 1983, doi: 10.1002/cyto.990040213.

[8] H. Edelsbrunner and E. P. Mücke, "Three-dimensional alpha shapes," ACM Trans. Graph., vol. 13, no. 1, pp. 43–72, 1994.

[9] C. T. Loop, "Smooth subdivision surfaces based on triangles."

[10] S. Thulung, K. Ranabhat, S. Bishokarma, and D. N. Gongal, "Morphometric Measurement of Cranial Vault Thickness: A Tertiary Hospital Based Study," J. Nepal Med. Assoc., vol. 57, no. 215, 2019.

[11] J. Kretschmer, G. Soza, C. Tietjen, M. Suehling, B. Preim, and M. Stamminger, "ADR - Anatomy-Driven Reformation," IEEE Trans. Vis. Comput. Graph., vol. 12, no. 12, pp. 2496–2505, 2014.
